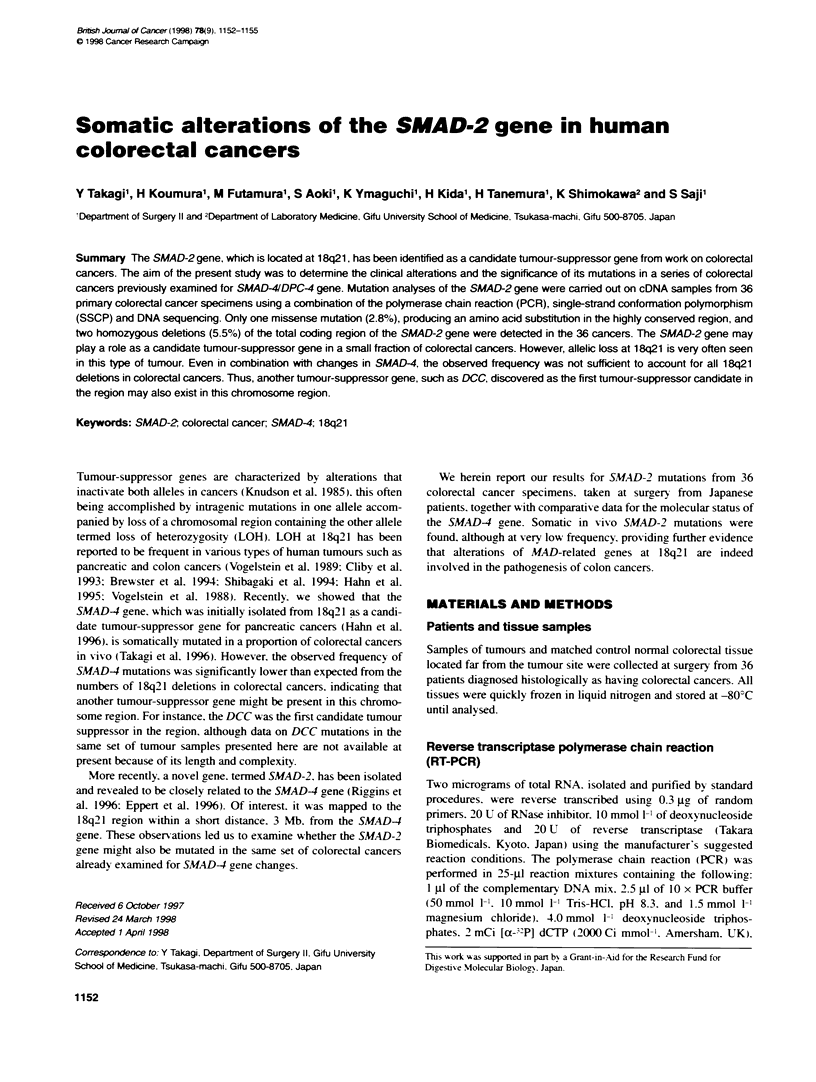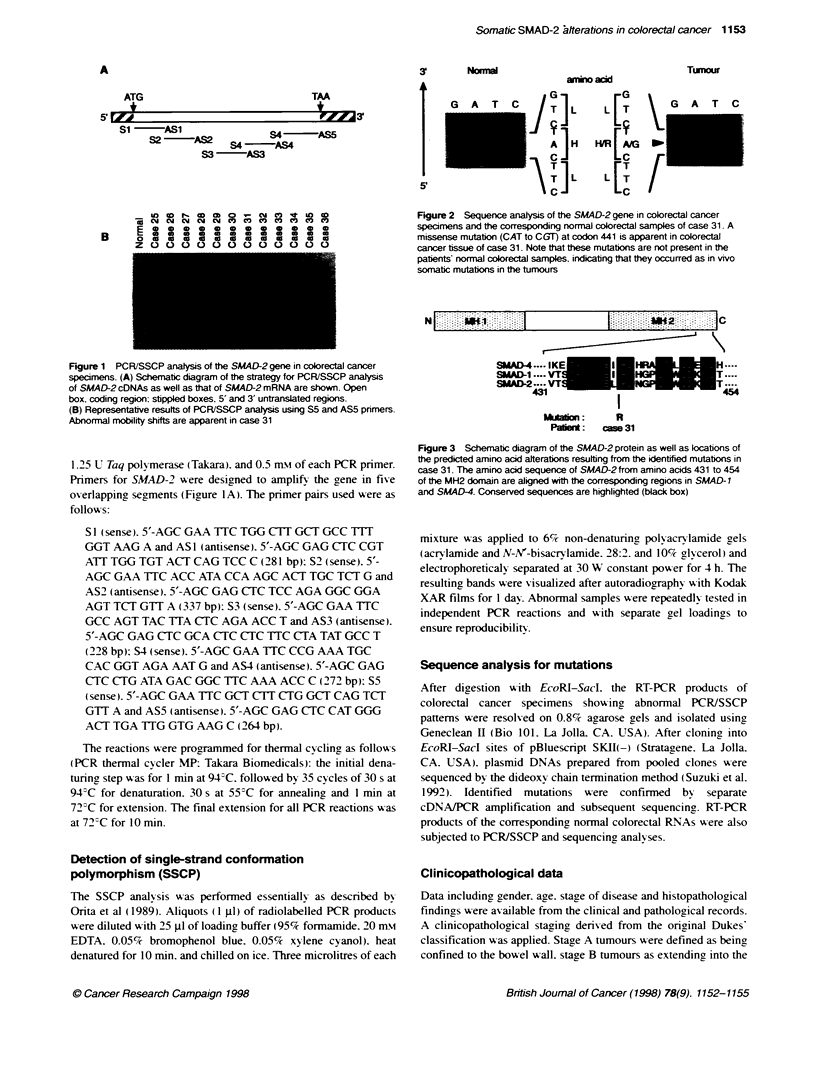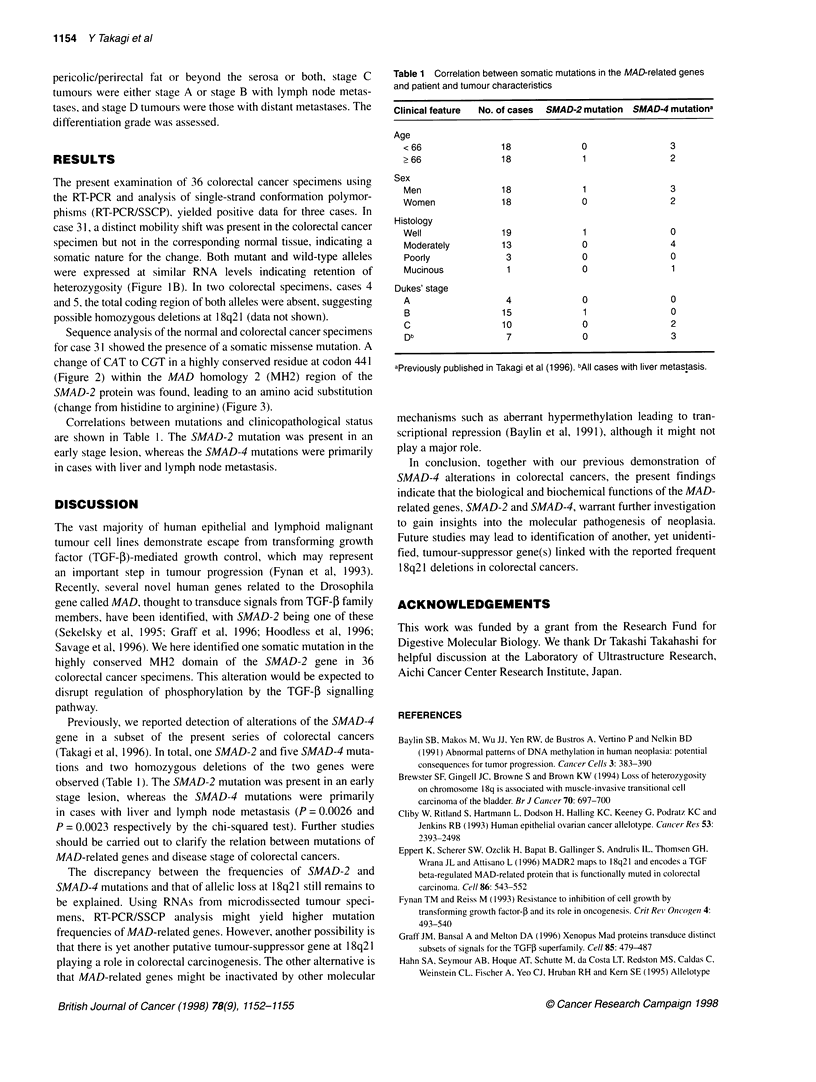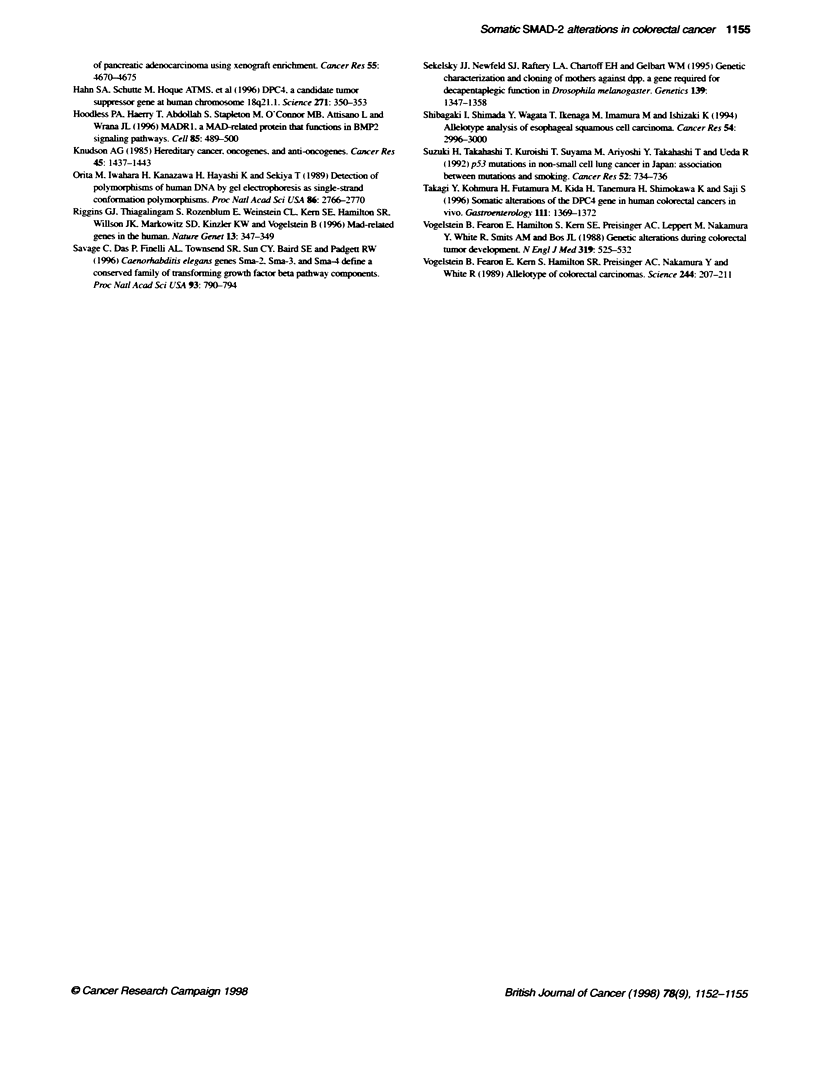# Somatic alterations of the SMAD-2 gene in human colorectal cancers.

**DOI:** 10.1038/bjc.1998.645

**Published:** 1998-11

**Authors:** Y. Takagi, H. Koumura, M. Futamura, S. Aoki, K. Ymaguchi, H. Kida, H. Tanemura, K. Shimokawa, S. Saji

**Affiliations:** Department of Surgery II, Gifu University School of Medicine, Japan.

## Abstract

**Images:**


British Journal of Cancer (1998) 78(9), 1152–1155
© 1998 Cancer Research Campaign

### Somatic alterations of the *SMAD-2* gene in human colorectal cancers

**Y Takagi[1], H Koumura[1], M Futamura[1], S Aoki[1], K Ymaguchi[1], H Kida[1], H Tanemura[1], K Shimokawa[2] and S Saji[1]**

[1]Department of Surgery II and [2]Department of Laboratory Medicine, Gifu University School of Medicine, Tsukasa-machi, Gifu 500-8705, Japan

**Summary** The *SMAD-2* gene, which is located at 18q21, has been identified as a candidate tumour-suppressor gene from work on colorectal cancers. The aim of the present study was to determine the clinical alterations and the significance of its mutations in a series of colorectal cancers previously examined for *SMAD-4/DPC-4* gene. Mutation analyses of the *SMAD-2* gene were carried out on cDNA samples from 36 primary colorectal cancer specimens using a combination of the polymerase chain reaction (PCR), single-strand conformation polymorphism (SSCP) and DNA sequencing. Only one missense mutation (2.8%), producing an amino acid substitution in the highly conserved region, and two homozygous deletions (5.5%) of the total coding region of the *SMAD-2* gene were detected in the 36 cancers. The *SMAD-2* gene may play a role as a candidate tumour-suppressor gene in a small fraction of colorectal cancers. However, allelic loss at 18q21 is very often seen in this type of tumour. Even in combination with changes in *SMAD-4*, the observed frequency was not sufficient to account for all 18q21 deletions in colorectal cancers. Thus, another tumour-suppressor gene, such as *DCC*, discovered as the first tumour-suppressor candidate in the region may also exist in this chromosome region.

**Keywords:** *SMAD-2*; colorectal cancer; *SMAD-4*; 18q21

Tumour-suppressor genes are characterized by alterations that inactivate both alleles in cancers (Knudson et al, 1985), this often being accomplished by intragenic mutations in one allele accompanied by loss of a chromosomal region containing the other allele termed loss of heterozygosity (LOH). LOH at 18q21 has been reported to be frequent in various types of human tumours such as pancreatic and colon cancers (Vogelstein et al, 1989; Cliby et al, 1993; Brewster et al, 1994; Shibagaki et al, 1994; Hahn et al, 1995; Vogelstein et al, 1988). Recently, we showed that the *SMAD-4* gene, which was initially isolated from 18q21 as a candidate tumour-suppressor gene for pancreatic cancers (Hahn et al, 1996), is somatically mutated in a proportion of colorectal cancers in vivo (Takagi et al, 1996). However, the observed frequency of *SMAD-4* mutations was significantly lower than expected from the numbers of 18q21 deletions in colorectal cancers, indicating that another tumour-suppressor gene might be present in this chromosome region. For instance, the *DCC* was the first candidate tumour suppressor in the region, although data on *DCC* mutations in the same set of tumour samples presented here are not available at present because of its length and complexity.

More recently, a novel gene, termed *SMAD-2*, has been isolated and revealed to be closely related to the *SMAD-4* gene (Riggins et al, 1996; Eppert et al, 1996). Of interest, it was mapped to the 18q21 region within a short distance, 3 Mb, from the *SMAD-4* gene. These observations led us to examine whether the *SMAD-2* gene might also be mutated in the same set of colorectal cancers already examined for *SMAD-4* gene changes.

*Received 6 October 1997*
*Revised 24 March 1998*
*Accepted 1 April 1998*

*Correspondence to:* Y Takagi, Department of Surgery II, Gifu University School of Medicine, Tsukasa-machi, Gifu 500-8705, Japan

We herein report our results for *SMAD-2* mutations from 36 colorectal cancer specimens, taken at surgery from Japanese patients, together with comparative data for the molecular status of the *SMAD-4* gene. Somatic in vivo *SMAD-2* mutations were found, although at very low frequency, providing further evidence that alterations of *MAD*-related genes at 18q21 are indeed involved in the pathogenesis of colon cancers.

### MATERIALS AND METHODS

### Patients and tissue samples

Samples of tumours and matched control normal colorectal tissue located far from the tumour site were collected at surgery from 36 patients diagnosed histologically as having colorectal cancers. All tissues were quickly frozen in liquid nitrogen and stored at –80°C until analysed.

### Reverse transcriptase polymerase chain reaction (RT-PCR)

Two micrograms of total RNA, isolated and purified by standard procedures, were reverse transcribed using 0.3 μg of random primers, 20 U of RNase inhibitor, 10 mmol $l^{-1}$ of deoxynucleoside triphosphates and 20 U of reverse transcriptase (Takara Biomedicals, Kyoto, Japan) using the manufacturer's suggested reaction conditions. The polymerase chain reaction (PCR) was performed in 25-μl reaction mixtures containing the following: 1 μl of the complementary DNA mix, 2.5 μl of 10 × PCR buffer (50 mmol $l^{-1}$, 10 mmol $l^{-1}$ Tris-HCl, pH 8.3, and 1.5 mmol $l^{-1}$ magnesium chloride), 4.0 mmol $l^{-1}$ deoxynucleoside triphosphates, 2 mCi [$\alpha$-$^{32}$P] dCTP (2000 Ci $mmol^{-1}$, Amersham, UK),

This work was supported in part by a Grant-in-Aid for the Research Fund for Digestive Molecular Biology, Japan.

**Figure 1** PCR/SSCP analysis of the *SMAD-2* gene in colorectal cancer specimens. (**A**) Schematic diagram of the strategy for PCR/SSCP analysis of *SMAD-2* cDNAs as well as that of *SMAD-2* mRNA are shown. Open box, coding region: stippled boxes, 5' and 3' untranslated regions. (**B**) Representative results of PCR/SSCP analysis using S5 and AS5 primers. Abnormal mobility shifts are apparent in case 31

**Figure 2** Sequence analysis of the *SMAD-2* gene in colorectal cancer specimens and the corresponding normal colorectal samples of case 31. A missense mutation (*CAT* to *CGT*) at codon 441 is apparent in colorectal cancer tissue of case 31. Note that these mutations are not present in the patients' normal colorectal samples, indicating that they occurred as in vivo somatic mutations in the tumours

**Figure 3** Schematic diagram of the *SMAD-2* protein as well as locations of the predicted amino acid alterations resulting from the identified mutations in case 31. The amino acid sequence of *SMAD-2* from amino acids 431 to 454 of the MH2 domain are aligned with the corresponding regions in *SMAD-1* and *SMAD-4*. Conserved sequences are highlighted (black box)

1.25 U *Taq* polymerase (Takara), and 0.5 mM of each PCR primer. Primers for *SMAD-2* were designed to amplify the gene in five overlapping segments (Figure 1A). The primer pairs used were as follows:

S1 (sense), 5'-AGC GAA TTC TGG CTT GCT GCC TTT GGT AAG A and AS1 (antisense), 5'-AGC GAG CTC CGT ATT TGG TGT ACT CAG TCC C (281 bp); S2 (sense), 5'-AGC GAA TTC ACC ATA CCA AGC ACT TGC TCT G and AS2 (antisense), 5'-AGC GAG CTC TCC AGA GGC GGA AGT TCT GTT A (337 bp); S3 (sense), 5'-AGC GAA TTC GCC AGT TAC TTA CTC AGA ACC T and AS3 (antisense), 5'-AGC GAG CTC GCA CTC CTC TTC CTA TAT GCC T (228 bp); S4 (sense), 5'-AGC GAA TTC CCG AAA TGC CAC GGT AGA AAT G and AS4 (antisense), 5'-AGC GAG CTC CTG ATA GAC GGC TTC AAA ACC C (272 bp); S5 (sense), 5'-AGC GAA TTC GCT CTT CTG GCT CAG TCT GTT A and AS5 (antisense), 5'-AGC GAG CTC CAT GGG ACT TGA TTG GTG AAG C (264 bp).

The reactions were programmed for thermal cycling as follows (PCR thermal cycler MP: Takara Biomedicals): the initial denaturing step was for 1 min at 94°C, followed by 35 cycles of 30 s at 94°C for denaturation, 30 s at 55°C for annealing and 1 min at 72°C for extension. The final extension for all PCR reactions was at 72°C for 10 min.

### Detection of single-strand conformation polymorphism (SSCP)

The SSCP analysis was performed essentially as described by Orita et al (1989). Aliquots (1 µl) of radiolabelled PCR products were diluted with 25 µl of loading buffer (95% formamide, 20 mM EDTA, 0.05% bromophenol blue, 0.05% xylene cyanol), heat denatured for 10 min, and chilled on ice. Three microlitres of each mixture was applied to 6% non-denaturing polyacrylamide gels (acrylamide and *N-N'*-bisacrylamide, 28:2, and 10% glycerol) and electrophoreticaly separated at 30 W constant power for 4 h. The resulting bands were visualized after autoradiography with Kodak XAR films for 1 day. Abnormal samples were repeatedly tested in independent PCR reactions and with separate gel loadings to ensure reproducibility.

### Sequence analysis for mutations

After digestion with *Eco*RI–*Sac*I, the RT-PCR products of colorectal cancer specimens showing abnormal PCR/SSCP patterns were resolved on 0.8% agarose gels and isolated using Geneclean II (Bio 101, La Jolla, CA, USA). After cloning into *Eco*RI–*Sac*I sites of pBluescript SKII(–) (Stratagene, La Jolla, CA, USA), plasmid DNAs prepared from pooled clones were sequenced by the dideoxy chain termination method (Suzuki et al, 1992). Identified mutations were confirmed by separate cDNA/PCR amplification and subsequent sequencing. RT-PCR products of the corresponding normal colorectal RNAs were also subjected to PCR/SSCP and sequencing analyses.

### Clinicopathological data

Data including gender, age, stage of disease and histopathological findings were available from the clinical and pathological records. A clinicopathological staging derived from the original Dukes' classification was applied. Stage A tumours were defined as being confined to the bowel wall, stage B tumours as extending into the pericolic/perirectal fat or beyond the serosa or both, stage C tumours were either stage A or stage B with lymph node metastases, and stage D tumours were those with distant metastases. The differentiation grade was assessed.

### RESULTS

The present examination of 36 colorectal cancer specimens using the RT-PCR and analysis of single-strand conformation polymorphisms (RT-PCR/SSCP), yielded positive data for three cases. In case 31, a distinct mobility shift was present in the colorectal cancer specimen but not in the corresponding normal tissue, indicating a somatic nature for the change. Both mutant and wild-type alleles were expressed at similar RNA levels indicating retention of heterozygosity (Figure 1B). In two colorectal specimens, cases 4 and 5, the total coding region of both alleles were absent, suggesting possible homozygous deletions at 18q21 (data not shown).

Sequence analysis of the normal and colorectal cancer specimens for case 31 showed the presence of a somatic missense mutation. A change of CAT to CGT in a highly conserved residue at codon 441 (Figure 2) within the *MAD* homology 2 (MH2) region of the *SMAD-2* protein was found, leading to an amino acid substitution (change from histidine to arginine) (Figure 3).

Correlations between mutations and clinicopathological status are shown in Table 1. The *SMAD-2* mutation was present in an early stage lesion, whereas the *SMAD-4* mutations were primarily in cases with liver and lymph node metastasis.

**Table 1** Correlation between somatic mutations in the *MAD*-related genes and patient and tumour characteristics

| Clinical feature | No. of cases | *SMAD-2* mutation | *SMAD-4* mutation[a] |
|---|---|---|---|
| Age | | | |
| < 66 | 18 | 0 | 3 |
| ≥ 66 | 18 | 1 | 2 |
| Sex | | | |
| Men | 18 | 1 | 3 |
| Women | 18 | 0 | 2 |
| Histology | | | |
| Well | 19 | 1 | 0 |
| Moderately | 13 | 0 | 4 |
| Poorly | 3 | 0 | 0 |
| Mucinous | 1 | 0 | 1 |
| Dukes' stage | | | |
| A | 4 | 0 | 0 |
| B | 15 | 1 | 0 |
| C | 10 | 0 | 2 |
| D[b] | 7 | 0 | 3 |

[a]Previously published in Takagi et al (1996). [b]All cases with liver metastasis.

### DISCUSSION

The vast majority of human epithelial and lymphoid malignant tumour cell lines demonstrate escape from transforming growth factor (TGF-β)-mediated growth control, which may represent an important step in tumour progression (Fynan et al, 1993). Recently, several novel human genes related to the Drosophila gene called *MAD*, thought to transduce signals from TGF-β family members, have been identified, with *SMAD-2* being one of these (Sekelsky et al, 1995; Graff et al, 1996; Hoodless et al, 1996; Savage et al, 1996). We here identified one somatic mutation in the highly conserved MH2 domain of the *SMAD-2* gene in 36 colorectal cancer specimens. This alteration would be expected to disrupt regulation of phosphorylation by the TGF-β signalling pathway.

Previously, we reported detection of alterations of the *SMAD-4* gene in a subset of the present series of colorectal cancers (Takagi et al, 1996). In total, one *SMAD-2* and five *SMAD-4* mutations and two homozygous deletions of the two genes were observed (Table 1). The *SMAD-2* mutation was present in an early stage lesion, whereas the *SMAD-4* mutations were primarily in cases with liver and lymph node metastasis ($P = 0.0026$ and $P = 0.0023$ respectively by the chi-squared test). Further studies should be carried out to clarify the relation between mutations of *MAD*-related genes and disease stage of colorectal cancers.

The discrepancy between the frequencies of *SMAD-2* and *SMAD-4* mutations and that of allelic loss at 18q21 still remains to be explained. Using RNAs from microdissected tumour specimens, RT-PCR/SSCP analysis might yield higher mutation frequencies of *MAD*-related genes. However, another possibility is that there is yet another putative tumour-suppressor gene at 18q21 playing a role in colorectal carcinogenesis. The other alternative is that *MAD*-related genes might be inactivated by other molecular mechanisms such as aberrant hypermethylation leading to transcriptional repression (Baylin et al, 1991), although it might not play a major role.

In conclusion, together with our previous demonstration of *SMAD-4* alterations in colorectal cancers, the present findings indicate that the biological and biochemical functions of the *MAD*-related genes, *SMAD-2* and *SMAD-4*, warrant further investigation to gain insights into the molecular pathogenesis of neoplasia. Future studies may lead to identification of another, yet unidentified, tumour-suppressor gene(s) linked with the reported frequent 18q21 deletions in colorectal cancers.

### ACKNOWLEDGEMENTS

This work was funded by a grant from the Research Fund for Digestive Molecular Biology. We thank Dr Takashi Takahashi for helpful discussion at the Laboratory of Ultrastructure Research, Aichi Cancer Center Research Institute, Japan.

### REFERENCES

Baylin SB, Makos M, Wu JJ, Yen RW, de Bustros A, Vertino P and Nelkin BD (1991) Abnormal patterns of DNA methylation in human neoplasia: potential consequences for tumor progression. *Cancer Cells* **3**: 383–390

Brewster SF, Gingell JC, Browne S and Brown KW (1994) Loss of heterozygosity on chromosome 18q is associated with muscle-invasive transitional cell carcinoma of the bladder. *Br J Cancer* **70**: 697–700

Cliby W, Ritland S, Hartmann L, Dodson H, Halling KC, Keeney G, Podratz KC and Jenkins RB (1993) Human epithelial ovarian cancer allelotype. *Cancer Res* **53**: 2393–2498

Eppert K, Scherer SW, Ozclik H, Bapat B, Gallinger S, Andrulis IL, Thomsen GH, Wrana JL and Attisano L (1996) MADR2 maps to 18q21 and encodes a TGF beta-regulated MAD-related protein that is functionally muted in colorectal carcinoma. *Cell* **86**: 543–552

Fynan TM and Reiss M (1993) Resistance to inhibition of cell growth by transforming growth factor-β and its role in oncogenesis. *Crit Rev Oncogen* **4**: 493–540

Graff JM, Bansal A and Melton DA (1996) Xenopus Mad proteins transduce distinct subsets of signals for the TGFβ superfamily. *Cell* **85**: 479–487

Hahn SA, Seymour AB, Hoque AT, Schutte M, da Costa LT, Redston MS, Caldas C, Weinstein CL, Fischer A, Yeo CJ, Hruban RH and Kern SE (1995) Allelotype

© Cancer Research Campaign 1998

of pancreatic adenocarcinoma using xenograft enrichment. *Cancer Res* **55**: 4670–4675

Hahn SA. Schutte M. Hoque ATMS. et al (1996) DPC4. a candidate tumor suppressor gene at human chromosome 18q21.1. *Science* **271**: 350–353

Hoodless PA. Haerry T. Abdollah S. Stapleton M. O'Connor MB. Attisano L and Wrana JL (1996) MADR1. a MAD-related protein that functions in BMP2 signaling pathways. *Cell* **85**: 489–500

Knudson AG (1985) Hereditary cancer. oncogenes. and anti-oncogenes. *Cancer Res* **45**: 1437–1443

Orita M. Iwahara H. Kanazawa H. Hayashi K and Sekiya T (1989) Detection of polymorphisms of human DNA by gel electrophoresis as single-strand conformation polymorphisms. *Proc Natl Acad Sci USA* **86**: 2766–2770

Riggins GJ. Thiagalingam S. Rozenblum E. Weinstein CL. Kern SE. Hamilton SR. Willson JK. Markowitz SD. Kinzler KW and Vogelstein B (1996) Mad-related genes in the human. *Nature Genet* **13**: 347–349

Savage C. Das P. Finelli AL. Townsend SR. Sun CY. Baird SE and Padgett RW (1996) *Caenorhabditis elegans* genes Sma-2. Sma-3. and Sma-4 define a conserved family of transforming growth factor beta pathway components. *Proc Natl Acad Sci USA* **93**: 790–794

Sekelsky JJ. Newfeld SJ. Raftery LA. Chartoff EH and Gelbart WM (1995) Genetic characterization and cloning of mothers against dpp. a gene required for decapentaplegic function in *Drosophila melanogaster*. *Genetics* **139**: 1347–1358

Shibagaki I. Shimada Y. Wagata T. Ikenaga M. Imamura M and Ishizaki K (1994) Allelotype analysis of esophageal squamous cell carcinoma. *Cancer Res* **54**: 2996–3000

Suzuki H. Takahashi T. Kuroishi T. Suyama M. Ariyoshi Y. Takahashi T and Ueda R (1992) *p53* mutations in non-small cell lung cancer in Japan: association between mutations and smoking. *Cancer Res* **52**: 734–736

Takagi Y. Kohmura H. Futamura M. Kida H. Tanemura H. Shimokawa K and Saji S (1996) Somatic alterations of the DPC4 gene in human colorectal cancers in vivo. *Gastroenterology* **111**: 1369–1372

Vogelstein B. Fearon E. Hamilton S. Kern SE. Preisinger AC. Leppert M. Nakamura Y. White R. Smits AM and Bos JL (1988) Genetic alterations during colorectal tumor development. *N Engl J Med* **319**: 525–532

Vogelstein B. Fearon E. Kern S. Hamilton SR. Preisinger AC. Nakamura Y and White R (1989) Allelotype of colorectal carcinomas. *Science* **244**: 207–211

© Cancer Research Campaign 1998